# A more complete picture of metal hyperaccumulation through next-generation sequencing technologies

**DOI:** 10.3389/fpls.2013.00388

**Published:** 2013-10-01

**Authors:** Nathalie Verbruggen, Marc Hanikenne, Stephan Clemens

**Affiliations:** ^1^Plant Physiology and Molecular Genetics, Bioengineering School, Faculty of Sciences, Université Libre de BruxellesBrussels, Belgium; ^2^Functional Genomics and Plant Molecular Imaging, Center for Protein Engineering, Department of Life Sciences, University of LiègeLiège, Belgium; ^3^PhytoSYSTEMS, University of LiègeLiège, Belgium; ^4^Department of Plant Physiology, University of BayreuthBayreuth, Germany; ^5^Bayreuth Center for Molecular Biosciences, University of BayreuthBayreuth, Germany

**Keywords:** hyperaccumulator, NGS, plant adaptation, genome-scan, RNA-Seq, epigenome, GWAS

## Abstract

The mechanistic understanding of metal hyperaccumulation has benefitted immensely from the use of molecular genetics tools developed for *Arabidopsis thaliana*. The revolution in DNA sequencing will enable even greater strides in the near future, this time not restricted to the family Brassicaceae. Reference genomes are within reach for many ecologically interesting species including heterozygous outbreeders. They will allow deep RNA-seq transcriptome studies and the re-sequencing of contrasting individuals to unravel the genetic basis of phenotypic variation. Cell-type specific transcriptome analyses, which will be essential for the dissection of metal translocation pathways in hyperaccumulators, can be achieved through the combination of RNA-seq and translatome approaches. Affordable high-resolution genotyping of many individuals enables the elucidation of quantitative trait loci in intra- and interspecific crosses as well as through genome-wide association mapping across large panels of accessions. Furthermore, genome-wide scans have the power to detect loci under recent selection. Together these approaches will lead to a detailed understanding of the evolutionary path towards the emergence of hyperaccumulation traits.

## INTRODUCTION

To maintain concentrations of essential metals within physiological limits and to cope with toxic non-essential metals, plants possess a sophisticated and tightly controlled metal homeostasis network that insures the balance between metal uptake, chelation, distribution and storage processes. A limited number of plant species (~500), the so-called metal hyperaccumulators, are able to colonize toxic soils heavily enriched or contaminated by metals, and display extraordinary leaf metal accumulation (Verbruggen et al., 2009; Krämer, 2010; Hanikenne and Nouet, 2011; van der Ent et al., 2013). Metal hyperaccumulators represent an extreme configuration of the metal homeostasis network. Understanding hyperaccumulation offers the promise of uncovering key nodes of the metal homeostasis network whose alterations can drastically modify metal accumulation and tolerance in plants. This knowledge could then be used to develop biotechnological tools for biofortification and phytoremediation strategies. Further, metal hyperaccumulators allow acquiring basic knowledge on the mechanisms underlying environmental adaptation and the evolution of complex traits.

Most of the current genetic, molecular and genomic data on metal hyperaccumulation have been obtained in *Arabidopsis halleri* and *Noccaea caerulescens* (for recent reviews, see Verbruggen et al., 2009; Krämer, 2010; Hanikenne and Nouet, 2011; Rascio and Navari-Izzo, 2011). The success of *A. halleri* and *N. caerulescens* as model systems essentially stems from the fact that they are close relatives of *Arabidopsis thaliana*. Research with these two model species has been greatly enhanced by taking advantage of the many resources developed for *A. thaliana, *including the availability of genetic maps, genome sequence, and commercial microarrays. Thus, molecular knowledge about metal hyperaccumulation and tolerance is currently limited both in terms of taxonomic diversity (*Brassicaceae*) and metal specificity (mostly Zn and Cd, and Ni for *N. caerulescens*).

Research on *A. halleri* and *N. caerulescens* has been following similar trends than in *A. thaliana* going from a candidate gene approach in a limited number of ecotypes to genome-wide studies in a vast range of natural accessions. However, it still lags behind because of the lack of reference genomes, limited potential to isolate mutants and the relatively low efficiency of plant transformation. We now have the opportunity to substantially deepen our understanding of *A. halleri* and *N. caerulescens. *Importantly, we can also go beyond these model species as the applications of next-generation sequencing technologies (NGS) will allow looking at a more diverse sampling of species outside the *Brassicaceae* (see other contributions in this issue). Tapping into natural diversity of plant phenotypes will reveal commonalities and differences in the adaptation of the metal homeostasis networks that support hyperaccumulation and tolerance.

**FIGURE 1:**
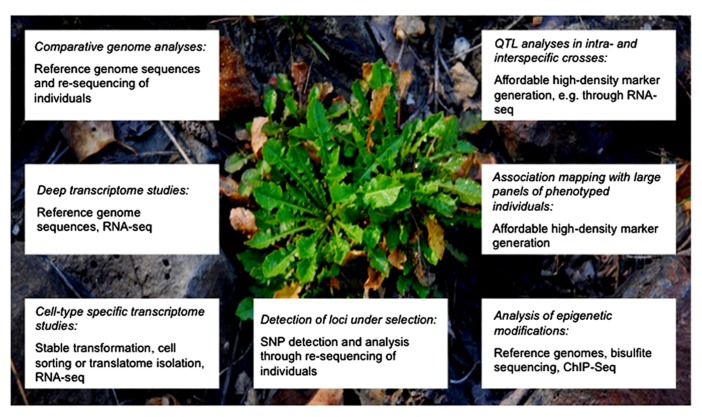
**Approaches enabled by NGS and expected to fundamentally advance the mechanistic understanding of metal hyperaccumulation.** Shown are different strategies (in italics) to unravel evolution, genetics and molecular physiology of metal hyperaccumulation. The lower panel in each box lists the main experimental techniques and resources required. The plant in the background is an *A. halleri* individual growing at a heavily Zn- and Pb-contaminated site. Image courtesy of Ricardo Stein, Romário Melo (University of Bochum) and Stephan Höreth (University of Bayreuth).

In this perspective note, we will highlight what can be expected from the use of NGS technologies to examine metal tolerance and accumulation mechanisms in hyperaccumulators (**Figure 1**). To simplify matters NGS refers here to all current and emerging high-throughput sequencing techniques. We will focus on *A. halleri* and *N. caerulescens* as examples, but as mentioned above, similar approaches will become tractable for many other species once reference genomes and transcriptomes become available. The feasibility of assembling a genome sequence *de novo* without prior genetic information using NGS has been demonstrated for *Thellungiella parvula* (Dassanayake et al., 2011).

## EVOLUTION OF THE HYPERACCUMULATION TRAIT

### EVOLUTION OF HYPERACCUMULATOR SPECIES

The selective advantage procured by hyperaccumulation is notoriously difficult to infer *a posteriori* and the driving force for the speciation of hyperaccumulators remains unclear. Non-metallicolous populations were shown to have evolved before colonization of metalliferous sites in *A. halleri* (Pauwels et al., 2005). It is thus postulated that the capacity to accumulate trace elements in the shoots afforded evolutionary advantage to *A. halleri* prior adaptation to contaminated soils. The most common hypothesis is the defense against herbivory and/or pathogens (see Boyd, 2007 and other contributions in this issue). Development of tools in population genomics will allow testing hypotheses and estimating the chronology of genetic changes underlying speciation and adaptation to metalliferous soils.

Zn hyperaccumulation and Cd hypertolerance seem to be constitutive traits in *A. halleri* and *N. caerulescens* at the species level (Verbruggen et al., 2009; Krämer, 2010; Hanikenne and Nouet, 2011). Both species present advantages as well as disadvantages when examining the genetic basis of those traits. As *A. halleri* is interfertile with *A. lyrata* ssp. *petraea*, species-specific traits have been analysed in segregating populations of interspecific crosses (Courbot et al., 2007; Willems et al., 2007, 2010; Frérot et al., 2010). As a self-incompatible outbreeder species, however, genome assembly may prove more difficult for *A. halleri* due to extensive heterozygosity. On the contrary, in *N. caerulescens*, genetic analyses are more difficult, because there is no interfertile related species that is Zn sensitive, Cd sensitive and a Zn non-accumulator, but as a self-pollinating species, *N. caerulescens* might be more amenable to genome assembly from NGS reads.

Comparative transcriptome studies of the two model species and related non-accumulators revealed a common set of metal homeostasis characteristics associated with constitutive hyperaccumulation traits. Several genes encoding metal transporters and enzymes involved in chelator biosynthesis are constitutively more strongly expressed in both hyperaccumulators (reviewed in Verbruggen et al., 2009; Krämer, 2010; Hanikenne and Nouet, 2011) indicating partially convergent evolution due to constraints of the metal homeostasis network (Hanikenne and Nouet, 2011).

Among the many highly expressed genes *in A. halleri,* classical genetic analysis, which provided a first view of the genetic architecture of hyperaccumulation traits, allowed also to pinpoint candidate genes with eventually the cloning and the validation of *HMA4*, a major gene for Zn and Cd tolerance and accumulation (Courbot et al., 2007; Hanikenne et al., 2008). *HMA4* encodes a Zn, Cd pump at the plasma membrane and is involved in metal translocation and redistribution (Hussain et al., 2004; Courbot et al., 2007; Hanikenne et al., 2008; Wong and Cobbett, 2009; Craciun et al., 2012). It represents an interesting case of convergent genetic evolution (Stern and Orgogozo, 2009). While *A. halleri* and *N. caerulescens *diverged at least 40 MY ago, *HMA4* is overexpressed in both species compared to non-hyperaccumulator species, due to gene tandem duplication and deregulated expression (Hanikenne et al., 2008; Ó Lochlainn et al., 2011; Craciun et al., 2012). Interestingly, Bayesian inference suggested that speciation between *A. halleri* and *A. lyrata*, which diverged much more recently than *A. halleri* and *N. caerulescens*, closely coincided with *HMA4* duplication (Roux et al., 2011).

Nevertheless, convergent phenotypic evolution, like hyperaccumulation, may not be associated with (completely) convergent genotypic evolution in plant genomes. Thurber et al. (2013) have for example shown that weedy (or red) rice has been selected for weed-adaptive traits through shared but also different genetic mechanisms. It is therefore interesting to investigate whether *HMA4* is also a hotspot gene in the adaptation of other Brassicaceae than *A. halleri* and *N. caerulescens* and of non-Brassicaceae Zn or Cd hyperaccumulators. More generally speaking, it will be important to determine to what extent the other differentially regulated metal homeostasis factors, e.g., *MTP1* (Dräger et al., 2004; Shahzad et al., 2010), *ZIP* or *NAS* genes (Krämer et al., 2007), are universally part of the apparent hyperaccumulation syndrome. In other words, this may shed light on the functional constraints in the metal homeostasis network. 

So far, hyperaccumulation has been assumed to rely essentially on an alteration of normal metal homeostasis but not on novel functions. The postulate is that genes involved in hyperaccumulation and hypertolerance are not species-specific or novel, but rather differently regulated, compared to non-hyperaccumulator species. This hypothesis will be verified when reference genomes are available, which will allow discovering species-specific genes and open the way for their functional characterization.

### INTRASPECIFIC VARIATION

The analysis of intraspecific natural variation in wild species has begun to elucidate the molecular bases of phenotypic differences in relation to local plant adaptation to distinct natural environments, and to determine the ecological and evolutionary processes that maintain this variation (Mitchell-Olds et al., 2007; Alonso-Blanco et al., 2009; Prasad et al., 2012).

While Zn hyperaccumulation and Cd hypertolerance seem to be constitutive in *A. halleri* and *N. caerulescens*, extensive intraspecific variation is observed for both traits (Verbruggen et al., 2009; Krämer, 2010; Hanikenne and Nouet, 2011). This applies even more to the non-constitutive hyperaccumulation characters (Cd, Ni). This pronounced intraspecific variation of metal tolerance and accumulation is associated with either different edaphic origins, in particular between metalliferous (i.e., metal contaminated) and non-metalliferous (i.e., non-contaminated) sites, or with phylogeographic patterns (Pauwels et al., 2005, 2012). In *A. halleri*, recent adaptations to anthropogenic metallic excess may have independently occurred within distinct phylogeographic units, which potentially have involved the evolution of a variety of genetic mechanisms (Pauwels et al., 2012). In this context, linking contrasting phenotypes to specific genetic changes should help identifying genes underlying variation and determining how the tolerance and hyperaccumulation traits evolved. Functional polymorphisms are known to appear in all types of genes and gene regions, and they may have multiple mutational causes: changes in coding or regulatory regions, epigenetics (see below). Environmental interactions (e.g., with microorganisms in the rhizosphere) may also account for part of the phenotypic variation (Rajkumar et al., 2009; Farinati et al., 2011).

A few genes have already been shown to potentially explain part of the differences between populations of *N. caerulescens*. Examples include *HMA3*, a tonoplastic pump involved in vacuolar metal sequestration (Ueno et al., 2011) and *HMA4* (Ó Lochlainn et al., 2011; Craciun et al., 2012). In both cases differences in expression levels between populations were related to differences in gene copy number. Thus, genes assumed to be involved in the evolution of the trait at the species level may also be accounting for intraspecific variation. Again, reference genome-enabled RNA-seq-based transcriptome studies of individuals with contrasting hyperaccumulation and/or hypertolerance will greatly facilitate the dissection of intraspecific variation and help determine whether this early conclusion can be generalized. NGS technologies also allow (i) direct comparison of inter-individual differences through the re-sequencing of genomes, and (ii) to accelerate genetic approaches as well as to increase their power.

While the time and expense required for the collection of genotype data were critical considerations in the past, the increasing availability of inexpensive DNA genotyping-by-sequencing methods (for instance, Elshire et al., 2011; Kumar et al., 2012) make genome-wide association (GWA) mapping of metal tolerance and accumulation a realistic method. GWA mapping using 349 *A. thaliana* accessions recently allowed identification of *HMA3* polymorphism as the main cause of natural variation in Cd leaf accumulation (Chao et al., 2012). Collections of *A. halleri* and *N. caerulesens* have been identified and partly already physiologically characterized (Reeves and Brooks, 1983; Meerts and Van Isacker, 1997; Escarré et al., 2000; Lombi et al., 2000; Bert et al., 2002; Assunção, 2003; Roosens et al., 2003). The number of individuals was however not sufficient for GWA studies. Ongoing efforts to establish larger genotype collections of *A. halleri* (U. Krämer and S. Clemens, unpublished results) and of *N. caerulescens* (M. Aarts, unpublished results) will provide a basis for GWA mapping and constitute invaluable material to address the evolution of hyperaccumulation.

Limitations of GWA studies can come from (i) population structure, that is, not all investigated individuals being equally distantly related to each other; (ii) allelic heterogeneity, that is, alleles at a single locus with similar effects on gene function having arisen repeatedly; or (iii) complex genetic architecture, where many different genes affect the same trait (Weigel, 2012). Statistical methods to control for population structure have been developed to reduce the inflation of false positive associations but an alternative is the complementary use of traditional linkage mapping in controlled crosses (Brachi et al., 2010 and references therein). Another possible limitation is that the extent of linkage disequilibrium is unknown for hyperaccumulators. It will be important to select germplasm that maximizes allelic diversity and the power to dissect hyperaccumulation traits. This will reveal the variation in the genetic architecture of the metal tolerance or accumulation traits and the existence of other cases of convergent evolution. NGS methods will in addition greatly facilitate genotyping (see Egan et al., 2012) and allow high genome coverage of accessions or progeny from intraspecific crosses, thereby improve the detection and molecular elucidation of QTLs. Currently Illumina HiSeq is the most widely used NGS platform but the read lengths are still relatively short (up to 150 bp). Other platforms have higher mean read length (for example Pacific Biosciences, Roche/454 GS FLX or Ion Torrent; Egan et al., 2012; Quail et al., 2012), which facilitate subsequent bioinformatics analysis.

### DETECTION OF LOCI UNDER SELECTION

In a genome-scan using a limited set of AFLP markers, Meyer et al. (2009) examined patterns of polymorphisms across metallicolous and non-metallicolous populations of *A. halleri* and identified loci under divergent selection that are linked either to the constitutive tolerance of the species or to the recent colonization of metalliferous sites. Linking allele frequencies across the genome with environmental conditions should allow identifying the specific alleles that were targets of selection, and selective forces that shaped the hyperaccumulator genomes during evolution. Such approaches will soon be greatly empowered when very large sets of single nucleotide polymorphisms (SNPs, for a recent review on SNP discovery through NGS see Kumar et al., 2012) become available through re-sequencing approaches. This is exemplified by a genome-wide *F*_ST_ (fixation index which estimates genetic differentiation among populations) scan based on whole genome re-sequencing of pooled *A. lyrata* individuals from distinct populations which allowed the identification of loci associated with the adaptation to serpentine soils (Turner et al., 2010).

Detailed analyses of nucleotide sequence diversity of (candidate) genes involved in metal tolerance and accumulation across hyperaccumulator populations may as well reveal whether selection acted on these genes. For example, a population genetics analysis of nucleotide polymorphism patterns of *HMA4 *in *A. halleri* provided evidence for (i) positive selection on *cis*-regulatory sequences and/or copy number expansion resulting in a selective sweep and (ii) ectopic gene conversion in coding sequences of *HMA4*, together substantiating selection for increased gene dosage (Hanikenne et al., 2013).

## HIGHER RESOLUTION TRANSCRIPTOME ANALYSIS

For *A. halleri* and *N. caerulescens*, transcriptomic studies using *Arabidopsis* DNA chips have revealed the importance of metal transport and detoxification processes in hyperaccumulation (see above, Krämer et al., 2007). The application of NGS to RNA-seq provides much deeper and more precise expression analysis. At least 20% of plant genes undergo alternative splicing, in which a single pre-mRNA can be processed into diverse transcripts, often encoding protein isoforms with distinct or even antagonistic functions (Egan et al., 2012). NGS RNA-seq enables to study splicing on a whole genome scale and assess how splicing may differ in hyperaccumulators and non-hyperaccumulators.

Read mapping to the *A. thaliana* (for *A. halleri* and *N. caerulescens*) or *A. lyrata* (for *A. halleri*) genomes and *de novo* transcriptome assemblies will provide very valuable resources. Moreover, the *A. halleri* and *N. caerulescens* genomes will soon become available enabling even more powerful analyses. Cross-species comparison of gene expression using RNA-seq, allowing to compare mechanisms of adaptation, is more challenging than within-species analyses. The availability of reference genomes/transcriptomes for each species combined with a clear picture of orthology relationships between genes should allow this using standard normalization procedures.

However, global transcriptomic approaches fail to provide a picture of the tissue-specific complexity. Metal accumulation is expected to vary between tissues and hence analysis at the organ level may mask contrasted expression levels or the role of specialized cell types. Organ-level analysis of transcriptomes is thus insufficient to dissect the network of metal chelator and transporter activities that mediates high fluxes and efficient hyperaccumulation of certain metals while maintaining homeostasis of other metals such as Fe, Cu, or Mn, whose trafficking pathways are at least partly shared with those of the hyperaccumulated metals. The importance of cell-type specific analysis is immediately evident also from the fact that metals are differentially accumulated in different cell types, e.g., mesophyll vs. epidermis cells in leaves of hyperaccumulators (White and Broadley, 2011). Furthermore, work in *A. thaliana* has clearly demonstrated the existence of cell type-specific transcriptional stress responses (Dinneny et al., 2008). For instance, transcriptome analysis with cellular resolution suggested a central role of genes expressed in the pericycle in controlling Fe deficiency responses, some of which are directly dependent on metal hyperaccumulation candidate genes such as *FRD3* and *NAS4* (Long et al., 2010).

With the exception of labor-intensive laser capture microdissection, current methods for cell type-specific analysis require stable transformation (Long, 2011). They are all based on the use of promoters driving gene expression specifically in distinct cell types. Fluorescence-activated cell sorting (FACS) uses specific labeling of cell types through the expression of fluorescent proteins (Birnbaum et al., 2003). Targeted affinity-purification of nuclei is enabled by the expression of a biotinylated nuclear envelope protein (INTACT) and allows mRNA and chromatin analysis (Deal and Henikoff, 2011; Bailey-Serres, 2013). The latter method, as well as the isolation of polysomal RNA via the expression of a tagged ribosomal protein (TRAP; Mustroph et al., 2009) to analyze the translatome, i.e., the actively translated mRNAs, circumvent the problems associated with protoplasting prior to isolation. On the other hand, unlike FACS these methods do not permit downstream analyses of cellular proteomes or metabolomes.

Stable transformation of hyperaccumulators has to date been successfully developed and applied only in *A. halleri* (Hanikenne et al., 2008; Deinlein et al., 2012). Provided gains in transformation efficiency are achieved the methods developed for *A. thaliana* can be directly adopted including the use of *A. thaliana* promoters since they are very likely to work in a similar fashion in the relative *A. halleri*. Application of these methods for hyperaccumulator species phylogenetically distant from *A. thaliana* will additionally require the isolation of suitable promoters.

Finally, it is evident that transcriptomics data need to be validated at the protein level. It is well known from studies in model organisms like *S. cerevisiae* that expression of the transcriptome and proteome are poorly correlated (Gygi et al., 1999; Beyer et al., 2004). Correlations between transcript or protein abundance and the trait (metal tolerance and/or abundance) eventually need to be validated by functional studies as for *HMA4* (Hanikenne et al., 2008) and *NAS2* (Deinlein et al., 2012) in *A. halleri*.

## EPIGENOME

Eukaryotic DNA is associated with proteins to form chromatin whose modification affects transcription. The epigenome corresponds to modified DNA and chromatin states that do not affect the DNA sequence. This includes DNA methylation, histone modification and nucleosome density (Cedar and Bergman, 2009; Hamilton and Robin Buell, 2012). Changes of epigenetics marks integrate both developmental and environmental cues, which regulate gene expression and ultimately create cell-specific or stimulus-specific expression patterns.

Genome-wide assessment of the cytosine methylation status can be achieved by treating genomic DNA with sodium bisulfite (BS) that converts unmethylated cytosine residues into uracils (Cokus et al., 2008; Lister et al., 2008, 2009; Becker et al., 2011). The BS-treated DNA with unchanged methylated cytosines can be deep-sequenced and the reads mapped on a reference genome (Chen and Pellegrini, 2012). Histone modifications can be analyzed by chromatin immunoprecipitation coupled to sequencing (ChIP-Seq; Furey, 2012). The relatively low cost of sequencing coupled with the ability for assembly of the (epi)genome and the transcriptome as well as quantification of transcript abundances allows now to investigate, for example, whether environmental exposure to metals alters the epigenome and how epigenetic status determines gene expression. A growing body of evidence indicates that environmental factors alter epigenetic marks, which in turn changes gene expression and results in new phenotypes (for reviews, see Brower, 2011; Herceg and Vaissière, 2011; Feil and Fraga, 2012). For example, transcriptomic heat response in *A. thaliana* is controlled by an alternative histone (Kumar and Wigge, 2010). Although it has been poorly examined so far, alteration of epigenetic gene regulation might thus represent an important source of phenotypic plasticity in plant stress responses (Mirouze and Paszkowski, 2011), in particular in response to environmental exposure to toxic metal concentrations.

## CONCLUSIONS

Using combined genomics, population genetics and quantitative genetics approaches (**Figure 1**) allow deepening our understanding of several essential biological and evolutionary questions that have been addressed in this note:

(i) Do functional constraints limit the adaptation of metal homeostasis networks in metal hyperaccumulators and what is the extent of convergent evolution? Does looking at a more diverse set of taxa will allow discovering alternative evolutionary paths?
(ii) Does the evolution of hyperaccumulation require novel gene functions?
(iii) What is the extent of interference and independence of pathways for the different metals in hyperaccumulators?
(iv) Is ecological adaptation to toxic soils at the onset of speciation?
(v) What are the mechanisms underlying intraspecific variation of tolerance and accumulation in hyperaccumulators?
(vi) What is the cellular specificity of gene expression and how does it correlate with protein abundance?

## Conflict of Interest Statement

The authors declare that the research was conducted in the absence of any commercial or financial relationships that could be construed as a potential conflict of interest.
